# Shedding Light on Specificity: Population Genomic Structure of a Symbiosis Between a Coral Reef Fish and Luminous Bacterium

**DOI:** 10.3389/fmicb.2019.02670

**Published:** 2019-11-19

**Authors:** Alison L. Gould, Paul V. Dunlap

**Affiliations:** ^1^Department of Ichthyology, California Academy of Sciences, San Francisco, CA, United States; ^2^Department of Ecology and Evolutionary Biology, University of Michigan, Ann Arbor, MI, United States

**Keywords:** RADseq, symbiont transmission, endosymbiosis, mutualism, *Photobacterium*

## Abstract

All organisms depend on symbiotic associations with bacteria for their success, yet how these interspecific interactions influence the population structure, ecology, and evolution of microbial symbionts is not well understood. Additionally, patterns of genetic variation in interacting species can reveal ecological traits that are important to gene flow and co-evolution. In this study, we define patterns of spatial and temporal genetic variation of a coral reef fish, *Siphamia tubifer*, and its luminous bacterial symbiont, *Photobacterium mandapamensis* in the Okinawa Islands, Japan. Using restriction site-associated sequencing (RAD-Seq) methods, we show that populations of the facultative light organ symbiont of *S. tubifer* exhibit genetic structure at fine spatial scales of tens of kilometers despite the absence of physical barriers to dispersal and in contrast to populations of the host fish. These results suggest that the host’s behavioral ecology and environmental interactions between host and symbiont help to structure symbiont populations in the region, consequently fostering the specificity of the association between host generations. Our approach also revealed several symbiont genes that were divergent between host populations, including *hfq* and a homolog of *varS*, both of which play a role in host association in *Vibrio cholerae*. Overall, this study highlights the important role that a host animal can play in structuring the distribution of its bacterial symbiont, particularly in highly connected marine environments, thereby promoting specificity of the symbiosis between host generations.

## Introduction

In open marine environments, where there are few physical barriers to dispersal, microbes are expected to exist as well-mixed populations, supporting the classic panmictic view in microbial biogeography that “everything is everywhere, but the environment selects” (Baas-Becking, 1934). However, this view has been challenged in recent years, providing evidence of geographic structure in certain marine microbes (Martiny et al., 2006; Ivars-Martínez et al., 2008), although often over broad geographic scales and with coarse taxonomic resolution (Pommier et al., 2005, 2007; Brown et al., 2012; Sul et al., 2013). In contrast, there are few studies that have quantitatively examined the spatial scale of intra-specific patterns of distribution in marine bacteria with sufficient fine-scale genetic resolution to differentiate populations (Acinas et al., 2004; DeChaine et al., 2006; Hunt et al., 2008). We define the population genomic structure of a luminous marine bacterium that is the facultative symbiont of a coral reef fish over a relatively small geographic region and reveal the important role a host animal’s behavioral ecology can play in symbiont transmission and in structuring populations of its microbial symbiont in a highly connected ocean environment.

The evolution of a symbiotic association requires that the specific partnership between host and symbiont remains stable over host generations. Such specificity is maintained in vertically transmitted symbioses via the intimate transfer of symbiont from parent to offspring and commonly leads to co-speciation (Chen et al., 1999; Clark et al., 2000; Moran et al., 2003; Hosokawa et al., 2006). In contrast, the evolution of horizontally transmitted symbioses requires both the selection of a particular symbiont from the environmental pool and the maintenance of that relationship over host generations. Therefore, a lower level of host-symbiont specificity is expected for horizontally acquired symbioses than that of their vertical counterparts. Nevertheless, many such associations are highly specific (Cavanaugh, 1994; Nyholm and McFall-Ngai, 2004), indicating that mechanisms such as environmental filtering and the ecology or physiology of the host ensure that a certain symbiont is acquired from the environment by each new host generation.

Bioluminescent symbioses, especially those involving fish hosts, display a higher degree of specificity than what would be expected due to random symbiont acquisition from the marine environment (Dunlap et al., 2007; Kaeding et al., 2007). How such specificity is achieved and maintained remains poorly understood, but the ecology of the host can play an important role (Lee and Ruby, 1994; Nyholm et al., 2000; Visick and Ruby, 2006). Unfortunately, little is known of the ecology of most symbiotically luminous fishes, especially with respect to symbiont acquisition, a process that is challenging to study in nature due to the planktonic larval state of most fishes and their often deep or open water habitats (Herring and Morin, 1978). The symbiotically luminous cardinalfish, *Siphamia tubifer*, inhabits shallow coral reefs in the Indo-Pacific and has known life history attributes (Gould et al., 2016) and behavior (Gould et al., 2014) that make it an ideal luminous fish species with which to investigate the host’s role in symbiont acquisition and in maintaining specificity of the symbiosis.

The bioluminescent symbiosis between *S. tubifer* and the luminous bacterium, *Photobacterium mandapamensis*, is a mutualistic association in which the symbiont is provisioned in an abdominal light organ by the fish in exchange for light production by the bacteria. The host, which remains quiescent among the long spines of sea urchins during the daytime (Tamura, 1982; Gould et al., 2014), uses the bacterially produced light to illuminate its ventral surface while foraging at night, possibly for countershading against the moonlight or for attracting prey. The host’s light organ is colonized by the luminous bacteria during larval development, although the exact timing in the wild remains unknown. In culture, *S. tubifer* larvae are not receptive to colonization by *P. mandapamensis* until at least 8 days after having been released into the plankton (Dunlap et al., 2012). Therefore, the direct transfer of symbiont from parent to offspring is not possible, and acquisition of the symbiont from the environment by aposymbiotic larvae occurs some time prior to settlement, as the light organs of all juvenile *S. tubifer* collected from a reef have an established symbiont population. Several aspects of the initiation of the symbiosis remain undefined, including the timing and location of symbiont acquisition in the wild as well as the number of bacterial cells that initially colonize a light organ.

To gain a better understanding of the symbiont distribution dynamics and strain diversity both within a light organ and among *S. tubifer* hosts, we applied double digest restriction site-associated sequencing (ddRAD-Seq) methods (Peterson et al., 2012) and quantified the within- and among- population genetic variability of the luminous bacteria from *S. tubifer* light organs collected from coral reefs in the Okinawa Islands, Japan. With this approach, we identified hundreds of variant sites throughout the symbiont’s genome and analyzed geographic patterns across these sites to test the hypothesis that the luminous symbionts of *S. tubifer* within a reef site are more genetically similar to each other than they are to symbionts from other reefs. We also tested for evidence of population structure in the host fish and compared the patterns of genetic variability of the host to that of their symbiotic bacteria. We then analyzed the temporal stability of the light organ symbiont population at a single reef site over three consecutive years. The results of this study provide insight into the process of symbiont acquisition for this highly specific, bioluminescent association, and we discuss the results in the context of the host fish’s role in structuring symbiont populations in the region.

## Materials and Methods

### Sampling, DNA Extraction and Library Preparation

**FIGURE 1:**
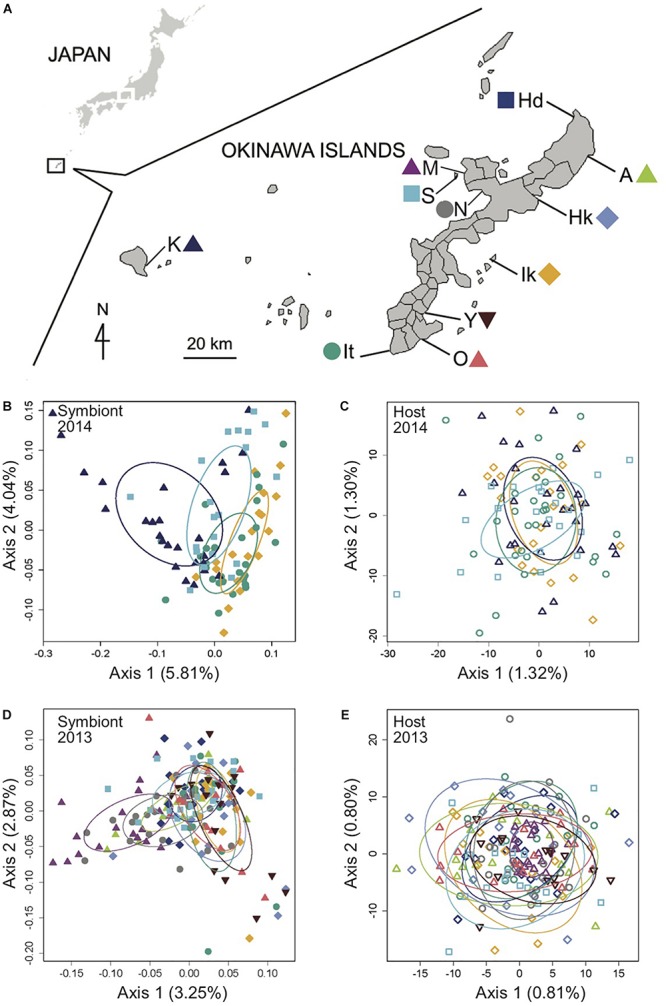
Genomic analysis of the light organ symbionts of *Siphamia tubifer* reveals structure in populations of symbiotic *Photobacterium mandapamensis* despite a lack of structure in the host fish. **(A)** Samples were collected from ten locations in 2013 and from four locations in 2014 in the Okinawa Islands, Japan. Principal coordinates analysis of genetic differentiation of **(B)** symbiotic *P. mandapamensis* across 534 loci analyzed from light organs sampled in 2014, **(C)** the corresponding *S. tubifer* hosts across 8,637 SNPs, **(D)** symbiotic *P. mandapamensis* across 465 loci analyzed from light organs sampled in 2013, and **(E)** the corresponding *S. tubifer* hosts across 8,637 SNPs. Points represent individuals along the first and second axes of genetic variation. The first 20 axes of variation for each analysis are depicted in Supplementary Figure S5. Ellipses indicate standard deviation for symbiont populations sampled at each location.

*Siphamia tubifer* specimens were collected over 3 years from locations in Okinawa, Japan (Table 1). Ten collection sites on Okinawa Island were sampled during June and July of 2013, and in June of 2014, samples were collected from an additional site on Kume Island as well as three sites on Okinawa Island that were previously sampled in 2013 (Figure 1A). One reef site (Sesoko, “S”) that was sampled in 2013 and 2014 was also sampled in July of 2012, providing a 3-year dataset from that location. All collection sites were less than 10 m depth. Approximately 20 fish ranging in body length were collected from several different urchins at each sampling location. Fish were immediately euthanized upon collection by immersion in seawater containing a lethal dose (500 mg/L) of buffered tricaine methanesulfonate (MS-222) as approved by the University of Michigan Institutional Animal Care and Use Committee.

**TABLE 1:** Location, year, and number of *Siphamia tubifer* light organs sampled for this study in the Okinawa Islands, Japan.

**ID**	**Location**	**Latitude**	**Longitude**	**Year**	***N***
S	Sesoko	26.635409	127.865832	2012	16
				2013	18
				2014	21
M	Motobu	26.655806	127.880286	2013	20
N	Nago	26.603673	127.932404	2013	21
Hd	Hedo	26.848756	128.252513	2013	17
It	Itoman	26.095109	127.658478	2013	14
				2014	27
O	Ou	26.127916	127.768981	2013	16
Y	Yonabaru	26.203032	127.771178	2013	16
Ik	Ikei	26.393535	127.988601	2013	15
				2014	22
Hk	Henoko	26.534554	128.046181	2013	17
A	Ada	26.741936	128.321107	2013	16
K	Kume	26.351617	126.820149	2014	26

The whole, intact light organ, composed of fish tissue and containing the luminous bacterial symbiont population, was aseptically removed from each fish specimen and individually preserved in RNAlater^®^. Genomic DNA was extracted from each light organ using a QIAGEN DNeasy Blood and Tissue Kit following the manufacturer’s protocol, and ddRAD-Seq libraries were built from the total DNA extracted from *S. tubifer* light organs, each individually barcoded with unique 10 bp DNA sequences for downstream identification. The protocol used to construct the libraries followed a modified combination of previously described methods (Parchman et al., 2012; Peterson et al., 2012), using the restriction enzymes *Mse*I and *Eco*RI, which were tested for their efficacy to produce a significant number of loci for the analysis of both the host fish and its symbiotic bacteria on an initial set of 50 light organs. Up to 50 DNA libraries were pooled per lane and sequenced at the Center for Applied Genomics, Toronto, ON, Canada, on the Illumina HiSeq2000 platform (San Diego, CA, United States), generating 100 bp single-end sequence reads.

### Sequence Processing and Data Analysis

Raw sequence reads were de-multiplexed and quality-filtered for a Phred score of 33 or higher with the *process_radtags* command in *Stacks* v1.35 (Catchen et al., 2011, 2013). After the DNA sequence barcodes were removed, the remaining 90-bp sequence reads were aligned against the ∼4.5 Mb reference genome of *P. mandapamensis* (Urbanczyk et al., 2011) using the *very_sensitive* command in *Bowtie2* (Langmead and Salzberg, 2012) v2.2.0 to separate the host fish sequences from those of its luminous symbiont. The unaligned sequences were additionally filtered against the genomes of the marine bacterium *Vibrio campbelli* (Lin et al., 2010) and *Escherichia coli* K12 (Durfee et al., 2008). All remaining unaligned sequences were classified as belonging to *S. tubifer*. Aligned *P. mandapamensis* sequences in *SAM* file format were then additionally quality filtered using *SAMtools* (Li et al., 2009) v1.3, retaining only reads with a MAPQ score greater than 20. The quality-filtered, aligned *P. mandapamensis* sequences were then processed with the *ref_map* command in *Stacks*, requiring a minimum stack depth of three (-m 3). Individuals with fewer than 100,000 total sequence reads or a mean depth of coverage per stack less than 100x were discarded from the analysis as they were considered to be outliers with respect to evenness of coverage across the individual sequence libraries (Supplementary Figure S1). The final dataset consisted of sequence reads from 282 light organs collected from 11 locations over 3 years in the Okinawa Islands (Table 1).

To minimize the effects of missing data, only bacterial loci present in at least 70% of all light organs were extracted from the entire catalog of loci produced by *Stacks* and used for the downstream analysis. Subsequently, loci not present in at least 50% of individuals in each population were removed from the dataset, resulting in an initial dataset of 601 RADtags (loci) across all individuals. Additional missing data filters were also tested but did not have a notable effect on the analysis (Supplementary Figure S2), thus we present the results for the missing data filters described above. After this initial filtering step, individuals sampled in 2013, 2014, and from Sesoko (S) over three consecutive years (2012–2014) were subsequently grouped into sub-datasets to analyze independently for genomic structure. To further minimize any potential effects of missing data within each dataset, both individuals and loci with more than 15% missing data were removed. The numbers of remaining loci analyzed were 465, 534, and 552 for the 2013, 2014, and Sesoko datasets, respectively. Within each locus, haplotypes with at least 6x coverage were then analyzed for patterns of genomic structure across light organ symbiont populations within each dataset. Additional haplotype depth filters (5x, 8x, and 10x) were also examined, but they also did not have a notable effect on the analysis (Supplementary Figure S3).

To visualize patterns of genetic variation in symbiont populations, principal coordinates analyses (PCoA) were performed on the Bray-Curtis dissimilarity matrices calculated from the presence or absence of these haplotypes across all light organs in each dataset. Significant differences in genetic differentiation were confirmed by a permutational multivariate analysis of variance (PERMANOVA) on the genetic dissimilarity matrices using the *adonis* function in the “vegan” (Oksanen et al., 2007) package in R (R Core Team, 2014) using sampling location (or year) as a factor. Similarly, pairwise *adonis* tests in “vegan” (Oksanen et al., 2007) were then performed between all pairs of locations within the 2013 and 2014 datasets (corrected for multiple comparisons) to determine which symbiont populations were significantly different from one another.

Patterns of genomic structure of the host fish were also analyzed and compared at the same scales as their light organ symbionts. *Siphamia tubifer* sequence reads were assembled *de novo* in *Stacks* and genotypes were assigned to each individual fish. Specifically, the first single nucleotide polymorphism (SNP) within each of the 8,637 loci present in 70% of individuals in each population with a minor allele frequency of 5% or greater were analyzed for each corresponding host fish. We selected these data filters based on previous RAD-Seq studies of other marine organisms (Benestan et al., 2015; Xu et al., 2017) in order to maximize the number of loci examined while minimizing the effects of missing data. Notably, the non-parametric analyses applied to these data tend to show little sensitivity to the minor allele filter (Linck and Battey, 2019). We then performed principal coordinates analyses on the Euclidean genetic distances between individuals in each of the *S. tubifer* SNP datasets (2013, 2014, and Sesoko) using the “adegenet” (Jombart and Ahmed, 2011) package in R. Weir and Cockerham’s pairwise *F*_*ST*_ values (Weir and Cockerham, 1984) between populations were calculated with 1,000 permutations in GenoDive, and corresponding *p*-values were adjusted for multiple comparisons using the Bonferroni method. To test for significant differences in genetic differentiation between sampling locations (or year) additional PERMANOVAs were performed on the genetic dissimilarity matrices as described above for the symbiotic bacteria. Mantel tests with 1,000 permutations were also performed on the dissimilarity matrices calculated for each dataset (2013, 2014, and Sesoko) to test for correlations between the genetic distances of the host fish to that of its light organ symbionts with the “vegan” package (Oksanen et al., 2007) in R.

To estimate the number of distinct symbiont strains present within a *S. tubifer* light organ, we determined the number of bacterial genotypes across all 601 loci within each host. One limitation of using RAD-Seq data to analyze symbiont populations is that haplotypes cannot be concatenated across loci, therefore we estimated the within light organ diversity to be the maximum number of haplotypes observed among all bacterial loci within each light organ. To determine whether symbiont diversity in a light organ increased over time, the number of symbiont genotypes present in each light organ was compared to the corresponding host fish’s standard length (a proxy for age; Gould et al., 2016).

**FIGURE 2:**
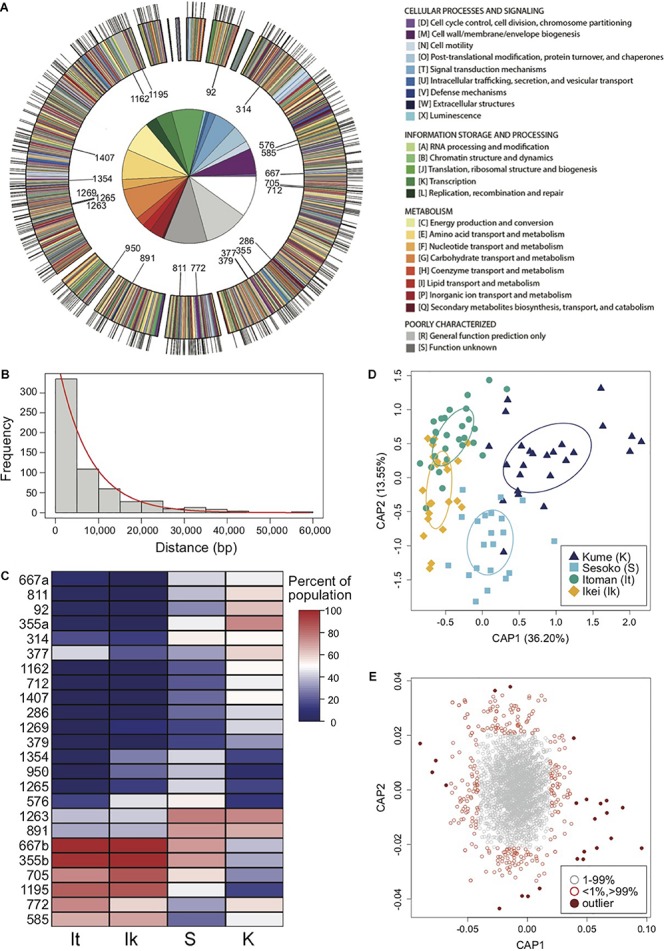
Restriction site-associated sequencing (RAD-Seq) yields a high density of genomic markers for the analysis of population structure and divergence of a symbiotic bacterium. **(A)** Annotated genome map of *Photobacterium mandapamensis*, the light organ symbiont of *Siphamia tubifer*. Protein-coding genes are color-coded according to which prokaryotic cluster of orthologous group (COG) they belong. Non-coding regions are white. Outer tic marks represent the locations of the 601 total loci analyzed for genomic differentiation between symbiont populations. The inner plot highlights the relative proportion of COG functions in which these loci are located. The 22 loci in which outlier haplotypes were identified as potential drivers of population differentiation are indicated on the inner circle of the map. **(B)** Distribution of the frequency of nucleotide distances between each pair of the 601 loci located in a scaffold of the *P. mandapamensis* genome. **(C)** Heatmap indicating the relative abundance within each population of the identified outlier haplotypes associated with each locus indicated. **(D)** Constrained analysis of principal coordinates (CAP) analysis of the 534 loci examined in 2014. Ellipses indicate standard deviation for symbiont populations sampled at each location. **(E)** Corresponding CAP scores of the haplotypes analyzed across all 534 loci.

To identify loci driving the patterns of genetic divergence between light organ symbionts at Kume Island and Okinawa Island in 2014, a constrained analysis of principal coordinates (CAP) using the *capscale* function in the “vegan” package (Oksanen et al., 2007) in R was performed on the 2014 *P. mandapamensis* genetic dissimilarity matrix. Haplotypes with the highest (>99%) and lowest (<1%) scores along first two axes of variation were identified and of these, 24 outlier haplotypes were further selected as potential outliers driving the observed genetic divergence between symbiont populations based on their large differences in CAP scores relative to all other haplotypes (Figures 2C–E and Supplementary Figure S4). For each of these outlier haplotypes, the associated locus and gene function were identified, and their variant type and functional effect were determined using the program *SnpEff* (Cingolani et al., 2012).

## Results

### Genetic Admixture of the Host Fish

We carried out an analysis of the RAD-Seq data for a total of 8,637 loci in 282 *S. tubifer* specimens sampled from eleven locations that were separated by two to tens of kilometers (local scale) around Okinawa Island and up to 140 km (regional scale) between Okinawa Island and Kume Island. The host fish exhibited no evidence of genetic structure at either scale sampled in 2013 and 2014 (Figure 1) and all pairwise *F*_*ST*_ values were extremely low, ranging between 0.001 and 0.005 (Supplementary Tables S1–S3), indicating that *S. tubifer* comprise one panmictic population in the region.

### Population Structure of the Bacterial Symbiont

Using a ddRAD-Seq approach, we identified a total of 601 bacterial loci for the light organ symbionts of *S. tubifer* across all 282 individuals. These loci were distributed approximately evenly throughout the *P. mandapamensis* genome (Figures 2A,B) and thus, provide a representative snapshot of the symbiont genome. Ordination analyses of the filtered subset of these loci (*n* = 534) from fish sampled in 2014 revealed a signature of genetic structure for populations of the symbiotic bacteria in contrast to the host fish (Figure 1), and was confirmed to be statistically significant by PERMANOVA (Table 2). This structure was particularly evident when comparing symbiont populations between Okinawa Island and Kume Island, located approximately 140 km to the West (Figure 1B), which featured symbionts that were genetically distinct from those sampled from the three locations on Okinawa Island (Supplementary Table S4). However, loci analyzed (*n* = 465) from light organs sampled in 2013 at the more local geographic scale of two to tens of kilometers suggest that the symbiotic bacteria are more genetically homogeneous around Okinawa Island (Figure 1D), although statistically significant differences between populations in Okinawa were also detected (Supplementary Table S5). Furthermore, no correlation in genetic distances was evident between the bacteria and their host fish for any of the three datasets (Mantel tests; 2013: *r* = −0.38, *p* = 1.00; 2014: *r* = −0.01, *p* = 0.53; Sesoko: *r* = −0.12, *p* = 0.89).

**TABLE 2:** Permutational multivariate analyses of variance (PERMANOVA) of the genetic distances between *Siphamia tubifer* hosts and between their light organ symbionts.

	**Host**					**Symbiont**				
**Dataset**	**DF**	**SS**	**F**	**R**	**P**	**DF**	**SS**	**F**	**R**	**P**
2014	3	15694	1.004	0.032	0.343	3	0.523	1.597	0.050	**<0.001**
2013	9	47367	1.008	0.054	0.131	9	1.099	1.249	0.064	**<0.001**
Sesoko	2	10718	1.027	0.038	**0.033**	2	0.225	1.092	0.043	0.093

*For each dataset the degrees of freedom (DF), sum of squares (SS), predicted *F*-value (F), R^2^ (R), and *P*-value are shown. For the 2014 and 2013 datasets, sampling location was used as the grouping factor, whereas sampling year was used for the Sesoko dataset. Significant *P*-values are in bold.*

### Loci Correlated With Symbiont Population Divergence

We identified putative loci driving the observed pattern of genetic divergence between populations of the symbiotic bacteria in 2014 and identified 24 outlier haplotypes from 22 loci. These haplotypes were highly correlated, either positively or negatively, with the primary axis of genetic variance differentiating the Kume Island and Okinawa Island populations (Figures 2C–E and Supplementary Figure S4). All 22 loci were located in protein coding regions of the genome, and most of the variant effects examined for each of the outlier haplotypes were non-synonymous, including two that resulted in the addition of a stop codon (Table 3).

**TABLE 3:** Outlier haplotypes for *Photobacterium mandapamensis* identified as potential drivers of genetic divergence between symbiotic populations at Kume and Okinawa Islands, Japan in 2014.

**ID**	**Scaffold**	**Variant Type**	**Variant Effect**	**Gene**	**Gene product**
92	2	Non-synonymous	Moderate	*hfq*	RNA chaperone Hfq
286	4	Synonymous	LOW	PMSV_1336	Unknown function
314	4	Synonymous	LOW	PMSV_145	Methyl-accepting chemotaxis protein
355a	4	Non-synonymous	Moderate	PMSV_1452	Efflux RND transporter permease subunit
355b		Consensus	-		
377	4	Non-synonymous	Moderate	*yeaG*	PrkA family serine protein kinase
379	4	Non-synonymous	Moderate	*yeaG*	PrkA family serine protein kinase
576	4	Upstream	Modifier	PMSV_554	Unknown function
585	4	Non-synonymous	Moderate	PMSV_564	Lipopolysaccharide assembly protein LapB
667a	4	Consensus	-	PMSV_772	Hsp70 family protein
667b		Stop gained	High		
705	4	Non-synonymous	Moderate	PMSV_851	Inosine/guanosine kinase
712	4	Non-synonymous	Moderate	VV12383	DUF2786 domain-containing protein
772	5	Non-synonymous	Moderate	*hpt*	Hypoxanthine phosphoribosyltransferase
811	5	Non-synonymous	Moderate	*barA*	Two-component sensor histidine kinase BarA
891	6	Non-synonymous	Moderate	PMSV_3308	Trk system potassium uptake protein
950	7	Non-synonymous	Moderate	PMSV_3736	Sigma-54-dependent Fis family transcriptional regulator
1162	8	Non-synonymous	Moderate	PMSV_2985	Unknown function
1195	8	Consensus	–	PMSV_3012	AsmA family protein
1263	8	Non-synonymous	Moderate	PMSV_2003	Alpha/beta fold hydrolase
1265	8	Stop gained	High	PMSV_2003	Alpha/beta fold hydrolase
1269	8	Synonymous	Low	PMSV_2013	Cobalamin ABC transporter substrate-binding protein
1354	8	Non-synonymous	Moderate	PMSV_2243	PTS fructose transporter subunit IIC
1407	8	Non-synonymous	Moderate	*gcvP*	Aminomethyl-transferring glycine dehydrogenase

*Listed are the locus ID assigned by *Stacks* (Catchen et al., 2011, 2013) corresponding to each haplotype, its variant type and effect as determined with *SnpEff*, and the putative gene product and category (COG) of the protein coding region in which each locus is located.*

### Temporal Stability of Symbiont Populations

**FIGURE 3:**
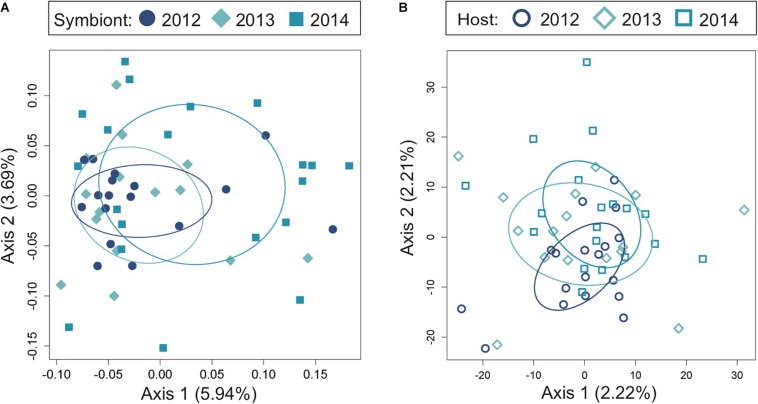
Temporal genomic analysis of the light organ symbionts of *Siphamia tubifer* reveals population stability at the same reef site over time. Light organs were sampled from reefs at Sesoko Island in Okinawa in the summer of 2012, 2013, and 2014. Principal coordinates analysis of genetic differentiation **(A)** of symbiotic *P. mandapamensis* across 552 loci and **(B)** of the corresponding *S. tubifer* hosts across 8,637 SNPs. Points represent individuals along the first and second axes of genetic variation. Ellipses indicate standard deviation for symbiont populations sampled each year.

To determine the extent to which light organ symbiont populations were stable over time, we also analyzed the genetic variance of bacteria from fish light organs collected from one location (Sesoko, Figure 1A) in three consecutive years (2012, 2013, and 2014). In contrast to the genetic differentiation observed between sampling locations in 2014, populations of the symbiotic bacteria were not significantly different between years at this location (Figure 3 and Table 2). This lack of variation suggests that the bacterial populations are somewhat stable at a reef over time. The host fish exhibited little genetic differentiation between years (Figure 3) although the PERMANOVA indicates that sampling year had a slight statistically significant effect on the genetic distances between the hosts sampled at Sesoko (Table 2).

### Within-Population Symbiont Strain Diversity

**FIGURE 4:**
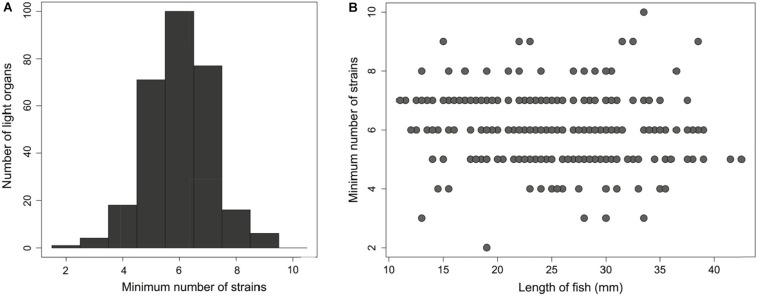
An average of six distinct *Photobacterium mandapamensis* strains are present within a light organ of *Siphamia tubifer* **(A)** Frequency distribution of the minimum possible number of symbiotic *P. mandapamensis* genotypes present within a light organ as determined by the maximum number of haplotypes observed across all 601 symbiont loci analyzed. **(B)** The minimum number of distinct *P. mandapamensis* strains present within a light organ relative to the standard length of the host fish.

In addition to defining the genomic diversity of symbiotic *P. mandapamensis* populations between reefs, we also examined symbiont diversity within individual light organs. Across all 282 *S. tubifer* specimens examined, an average of six (±1.6 SD) distinct bacterial genotypes were detected within a light organ (Figure 4), although this could be an underestimate of the total strain diversity present due to our inability to concatenate haplotypes across the 601 loci examined. Individual hosts had anywhere between two and ten symbiont types present in their light organ, and no fish harbored a population comprised entirely of a single symbiont genotype (Figure 4). Furthermore, there was no correlation between fish length, a proxy for fish age (Gould et al., 2016), and the minimum or maximum number of symbiont genotypes in a light organ (*F*_1_,_290_ = 1.477, *R^2^_*Adj*_* = 0.00164, *P* = 0.225) (Figure 4), indicating the number of strains within a light organ does not increase over time.

## Discussion

We obtained data for discrete, live populations of bacterial symbionts by extracting and sequencing genomic DNA directly from whole light organs of *S. tubifer*, a symbiotically luminous fish commonly found on coral reefs in the Okinawa Islands. This approach together with RAD-Seq methods allowed us to obtain deeply sampled, fine-scale genomic data capable of distinguishing populations of this bacterium from various locations sampled in the region, and in the absence of obvious barriers to dispersal. Although RAD-Seq methods have become increasingly popular in recent years (Rowe et al., 2011; Andrews et al., 2016), they have seldom been used to examine the genomic structure of natural populations of bacteria or to simultaneously examine the population structure of an animal host and its bacterial endosymbiont. By applying these methods, we were able to identify strain-level variation in the bacterial symbionts of *S. tubifer* populations sampled over a relatively small geographic region in the Okinawa Islands. Furthermore, we were able to identify potential genes of interest that appear to be divergent between symbiont populations.

Several of the variants identified were in genes of known importance for host association in species of symbiotic *Aliivibrio* and *Vibrio*, genera closely related to *Photobacterium*, including *hfq* (RNA-binding protein; Ding et al., 2004; Lenz et al., 2004), *barA* (histidine kinase; Lenz et al., 2005; Jang et al., 2011, Kamp et al., 2013), and *gcvP* (glycine dehydrogenase; Vanhove et al., 2017), and could therefore also be of importance for the bioluminescent association between *S. tubifer* and *P. mandapamensis*. Notably, there were non-synonymous variations in *barA* and *hfq* that were completely absent in the two of the four populations examined (Table 2 and Figure 2E). The two-component *barA/uvrY* homologs *varS/varA* in *Vibrio cholerae* have been recognized as important for virulence (Jang et al., 2011), quorum sensing (Lenz et al., 2005), and dissemination from a host to an aquatic environment (Kamp et al., 2013), and *varS* deletion strains of *V. cholerae* have colonization deficiencies in mice (Jang et al., 2011). Similarly, the RNA-binding protein Hfq is essential for the virulence of *V. cholerae*, and strains lacking *hfq* also fail to colonize mice hosts (Ding et al., 2004). *Hfq* has also been identified as a negative regulator of bioluminescence in *V. harveyi* (Lenz et al., 2004). Additional variants of interest were observed in *gcvP*, which plays a role in the virulence of *V. cholerae* in *Drosophila* hosts (Vanhove et al., 2017), and in *hpt*, which is located just upstream of the quorum-sensing regulating gene *luxR* in *V. harveyi* (Showalter and Silverman, 1990). The bacterial genes involved in the *S. tubifer*-*P. mandapamensis* symbiosis have yet to be identified and little is known regarding the genetics of this association, however our approach uncovered specific genes as interesting targets for future studies of the underlying mechanisms regulating this bioluminescent association.

Our genomic analysis of the host fish populations confirms previous findings that *S. tubifer* comprise one panmictic population in the Okinawa Islands likely due to larval dispersal (Gould and Dunlap, 2017) during their month-long pelagic stage (Gould et al., 2016). Northward flow of the Kuroshio Current from the Philippine Islands promotes connectivity of marine populations in the region (Rodriguez-Lanetty and Hoegh-Guldberg, 2002; Nishikawa and Sakai, 2005), and a high frequency of typhoons and heavy predation on adult fish (Gould et al., 2016) presumably all contribute to population turnover and the observed genetic admixture in the region. A previous analysis of outlier loci from this dataset revealed a strong signature of temporal differentiation likely due to variable larval supply from different upstream sources (Gould and Dunlap, 2017).

Despite the absence of physical barriers to dispersal and in contrast to the host fish, we discovered within-species level genetic differences between the luminous symbionts of *S. tubifer* sampled from different reefs in the Okinawa Islands. Furthermore, we determined that symbiont populations are seemingly stable at a reef between years. Environmental factors known to greatly influence the biogeography of marine bacteria include both temperature and salinity (Martiny et al., 2006). Fine scale differences in these and other variables between sites around Okinawa Island have previously been suggested to influence the distribution of different lineages of the algal symbiont of the zoantharian *Palythoa tuberculosa* (Noda et al., 2017) and could also influence the distribution of *P. mandapamensis* in the environment in this study. However, host organisms can also play a major role in the dispersal and geographic distribution of microbes in the marine environment (Troussellier et al., 2017). We suggest that the distinct behavioral ecology of *S. tubifer*, namely the fish’s homing and site fidelity behaviors (Gould et al., 2014), help shape the observed patterns of genetic structure between light organ symbionts in the Okinawa Islands.

The *S. tubifer* light organ is connected by a duct to the host’s intestine, and luminous bacterial cells are continually released via the duct from the light organ into the intestines and then the seawater with fecal waste (Dunlap and Nakamura, 2011). The release of bacterial cells from the light organ presumably promotes the growth and light production of the symbiont population inside the light organ while incidentally enriching the surrounding seawater with symbiont cells (Ruby et al., 1980; Haygood et al., 1984; Nealson et al., 1984; Lee and Ruby, 1994). Once settled as juveniles, *S. tubifer* exhibit fidelity to a home reef and typically return to the same urchin at that reef after foraging each night (Gould et al., 2014). Therefore, the *P. mandapamensis* genotypes present in the light organs of resident fish at a reef are regularly released into the surrounding seawater and can become enriched in the local environment. Thus, the observed genetic structure of *P. mandapamensis* symbionts could be a consequence of symbiont acquisition by larval fish occurring in the locally symbiont-enriched seawater near their settlement site. As a result, *S. tubifer* hosts residing at a particular reef, although not closely related, share more similar symbiont genotypes with each other than with fish from other reefs. This host-mediated symbiont population structure is consistent with the lack of temporal structure observed for symbiont populations sampled at the same reef over 3 years and has been observed for other marine symbioses (Taylor et al., 2005), including the bioluminescent symbiosis between *E. scolopes* and *A. fischeri* (Wollenberg and Ruby, 2009). Additionally, we did not see an increase in the number of symbionts present in a light organ with fish age, suggesting that colonization of a light organ occurs within a particular window of time by an average of six distinct *P. mandapamensis* strains, which then persist within the light organ throughout the host’s life.

In a highly connected ocean environment, where the classic view of microbial biogeography suggests that “everything is everywhere” (Baas-Becking, 1934), we illustrate that fine-scale patterns of genomic population structure in a facultative bacterial symbiont can arise, even in a region dominated by strong ocean currents and in the absence of physical barriers to gene flow. These results provide new insight on the timing and location of symbiont acquisition by *S. tubifer* larvae in the wild, a process that is difficult to study for most horizontally acquired marine symbioses. We hypothesize that the distinct life history and behavioral ecology of *S. tubifer* results in the local enrichment of *P. mandapamensis* in the surrounding seawater, which consequently ensures that the next generation of host fish can successfully initiate its symbiosis with its species-specific luminous bacterial symbiont. This host facilitation presumably helps to foster the high degree of specificity observed for the partnership (Kaeding et al., 2007) over time, and incidentally, can promote genetic divergence between symbiont populations. Future applications of RAD-Seq methods with other symbiotic associations have the potential to broaden our understanding of the influence of host animals on the biogeographic patterns of their microbial symbionts and the evolutionary processes that lead to host-symbiont integration and specificity.

## Data Availability Statement

The raw Illumina sequences from this study are accessible at NCBI SRA Accession No. SRP105806 (Biosample Accession Nos: SAMN06857385–SAMN06857664). Additional documents such as the STACKS catalogs and haplotype files generated will be made available by the authors, without undue reservation, to any qualified researcher.

## Ethics Statement

The animal study was reviewed and approved by The University of Michigan’s Institutional Animal Care and Use Committee.

## Author Contributions

AG conceived and designed the study, performed the laboratory work, and analyzed the data. AG and PD performed the fieldwork and wrote the manuscript.

## Conflict of Interest

The authors declare that the research was conducted in the absence of any commercial or financial relationships that could be construed as a potential conflict of interest.
